# Penetrative Laryngeal Cartilage Fracture: A Case Report

**DOI:** 10.7759/cureus.101041

**Published:** 2026-01-07

**Authors:** Robert Balsiger, Saketh Reddy Bathula, Michael D Mattin, Noah A Stern

**Affiliations:** 1 Otolaryngology – Head and Neck Surgery, Detroit Medical Center, Detroit, USA; 2 Otolaryngology - Head and Neck Surgery, University of Toledo College of Medicine and Life Sciences, Toledo, USA; 3 Otolaryngology, Ohio University Heritage College of Osteopathic Medicine, Athens, USA

**Keywords:** airway reconstruction, airway trauma, anterior commissure, laryngeal fracture, neck trauma, penetrating neck injury

## Abstract

Laryngeal fractures are rare but potentially life-threatening. Because the larynx plays a central role in airway patency, phonation, and airway protection, disruption of its structural integrity can result in severe morbidity. Early diagnosis is critical; however, due to the rarity of this injury and the wide spectrum of clinical presentation, laryngeal trauma is frequently underrecognized. When identified, management must prioritize airway stabilization, followed by anatomic reconstruction to optimize long-term voice and swallow outcomes. Laryngeal fractures from penetrating trauma demand rapid recognition and decisive airway management to prevent life-threatening compromise. Early surgical repair remains essential for optimizing long-term function. We successfully repaired a 60-year-old patient who presented to the emergency department with a gunshot wound to the laryngeal cartilage with anterior commissure disruption. The excellent functional recovery of the patient highlights the importance of early recognition, prompt airway intervention, and precise anatomic reconstruction, particularly in cases involving the anterior commissure.

## Introduction

Laryngeal fractures, though rare, represent a significant and potentially life-threatening injury, with an estimated incidence of approximately one in 30,000 trauma admissions [1]. The larynx plays a critical role in maintaining airway patency, phonation, and protection against aspiration, making disruption of its structural integrity particularly concerning. Traumatic injury to the larynx can result from a variety of mechanisms, including blunt trauma, penetrating trauma, and iatrogenic causes [2]. The clinical presentation of laryngeal fractures can be highly variable, ranging from subtle signs such as hoarseness and dysphagia to life-threatening airway compromise, which complicates early diagnosis [3].

Despite the importance of early recognition and intervention, traumatic laryngeal injuries are often underrecognized due to the spectrum of symptoms and the lack of specific diagnostic markers. Common sequelae of untreated or poorly managed laryngeal trauma include chronic dysphonia, dysphagia, aspiration, and in some cases, long-term dependence on tracheostomy [4]. Given the potential for significant morbidity, timely diagnosis and intervention are crucial. Early identification can be achieved through a thorough clinical evaluation, including a high index of suspicion in patients with signs of airway distress, hoarseness, or difficulty swallowing following neck trauma [5].

Management of laryngeal fractures involves two primary objectives: airway stabilization and anatomic reconstruction. Airway stabilization is paramount, especially in cases where the integrity of the airway is compromised, and may require intubation or a tracheostomy to ensure patency. Once the airway is secure, surgical or conservative management is employed to restore the larynx's structural and functional integrity. In cases of significant fracture or dislocation, surgical intervention is often required to ensure optimal long-term outcomes in voice and swallow function. Conservative management, including close observation, is appropriate for less severe injuries where the fracture does not threaten the airway [6].

This case report aims to highlight the complexities of managing traumatic laryngeal fractures and the importance of adhering to established guidelines in the diagnosis and treatment of such injuries. We present a case of traumatic laryngeal injury, review the diagnostic and management strategies employed, and discuss the long-term outcomes following treatment.

## Case presentation

A 60-year-old male patient presented emergently to the emergency department with two gunshot wounds. The first gunshot wound was at the right anterior neck in zone two at the level of the cricothyroid. The second gunshot wound was at the angle of the mandible to the anterior chest at the sternoclavicular joint.

The skin and mucus membranes were pale in color with a glass glaucoma scale of 13. Blood pressure was 117/79 mmHg (reference value: <120/80 mmHg), and pulse was 94 beats per minute (reference range: 60-100 bpm). The respiratory rate was 14 breaths per minute (reference range: 12 to 20 breaths per minute). Schafer's classification for laryngeal fracture [7] was group three. His respiratory status was compromised due to diffuse crepitus overlying the neck and chest. The emergency room physician decided to intubate the patient to secure the airway. Oral intubation via GlideScope was attempted three times by the emergency physician and the anesthesiologist; however, these attempts were unsuccessful due to active bleeding and laryngeal trauma. Emergent cricothyroidotomy was performed, and the patient's airway was secured by the trauma surgery team. He was then transferred to the operating room (OR) for standard tracheostomy. 

**Figure 1:**
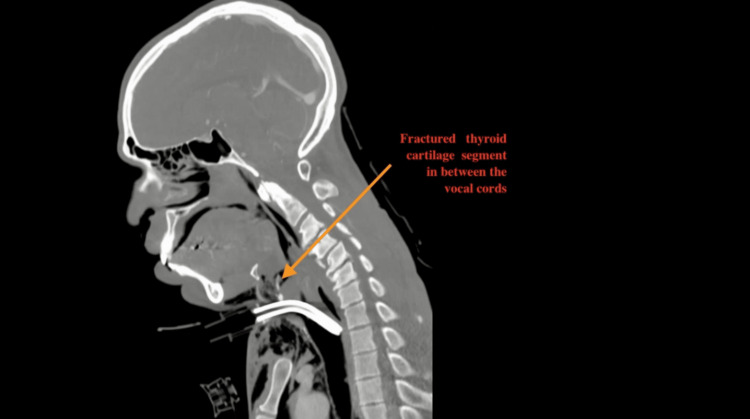
Pre-operative neck CT scan (sagittal view) The arrow shows the fractured thyroid cartilage segment separated from the thyroid cartilage.

**Figure 2:**
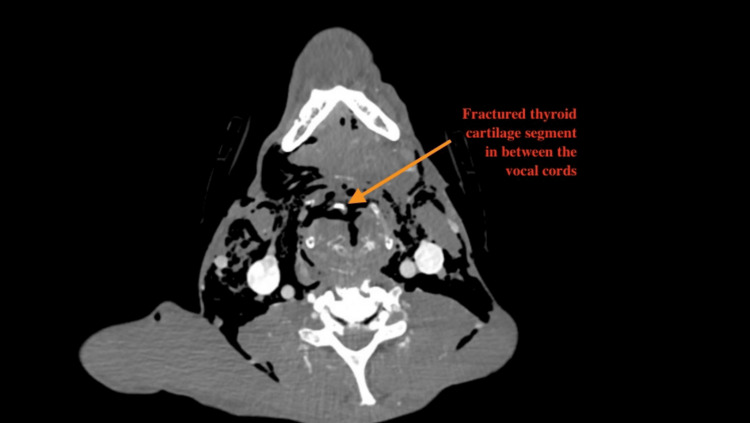
Pre-operative neck CT scan (axial view) The arrow indicates the fractured thyroid cartilage segment separated from the thyroid cartilage.

Computed Tomography (CT) of the head, neck, and chest with contrast showed a comminuted anterior thyroid cartilage fracture with diffuse subcutaneous emphysema as well as a left bicortical mandibular ramus fracture (Figures 1, 2).

The CT also showed a fracture of the left hyoid bone. There were no other cervical spine or soft tissue injuries. There were bilateral pulmonary contusions.

Surgical procedure

**Figure 3:**
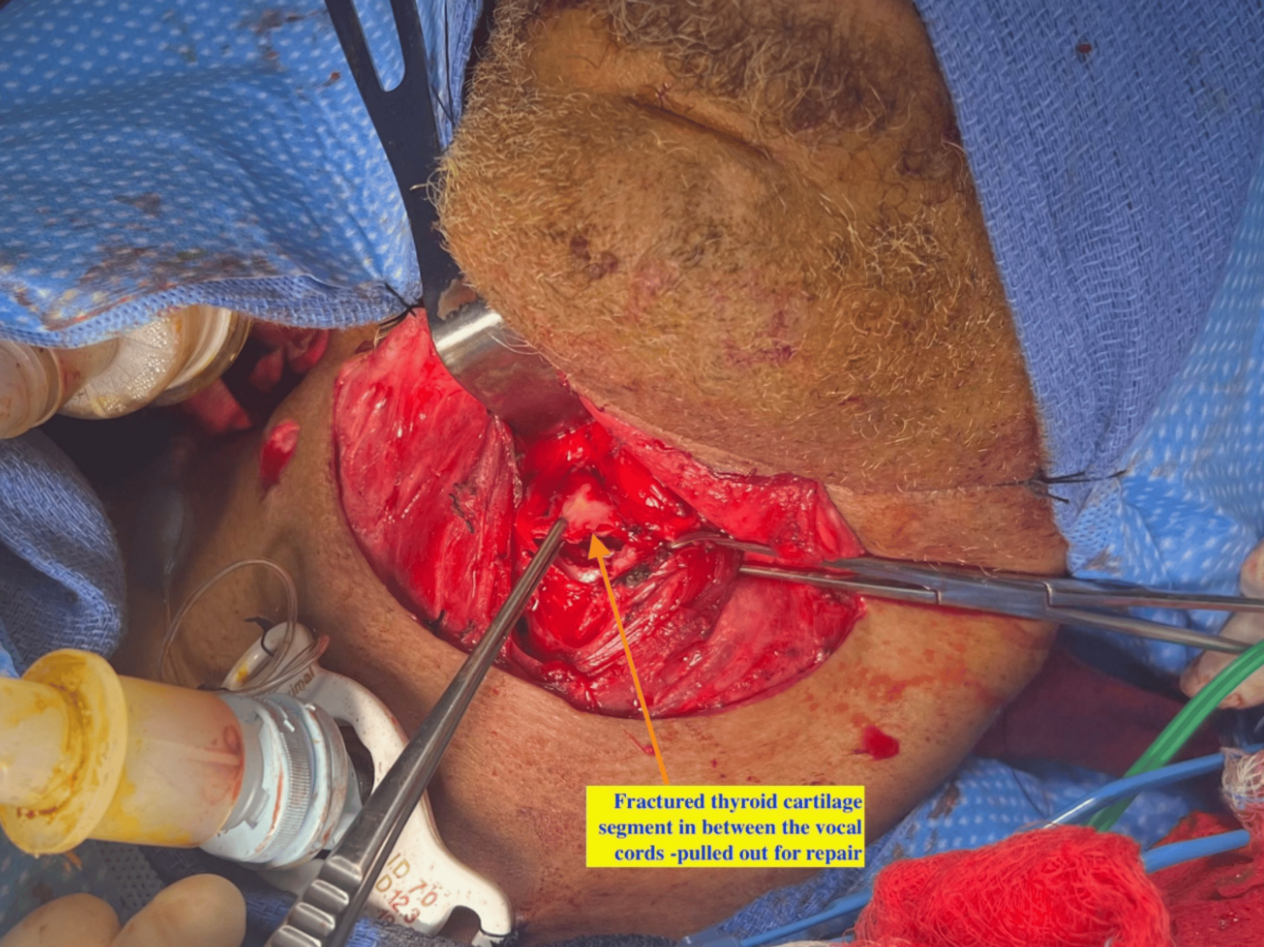
Intra-operative picture of fractured thyroid cartilage segment separated from the thyroid cartilage

After neck exploration, there was an appreciable fracture at the anterior aspect of laryngeal cartilage with completely mobile segment and communication of supraglottis as shown in the pre-operative CT scan. Intraoperatively, the Broyle's ligament was found to be completely disrupted (Figure 3).

The right and left vocal fold showed a loss of anterior attachment. The left and right vocal folds were secured anteriorly to the lateral aspect of the laryngeal perichondrium with horizontal mattress 4-0 Vicryl (Ethicon, Somerville, NJ, US). The thyrohyoid ligament was reapproximated posterior to laryngeal cartilage with 4-0 Vicryl. The freely mobile fractured segment of laryngeal cartilage was highly ossified making suture placement difficult and challenging. A 2-0 Prolene suture (Ethicon, Somerville, NJ, US) was placed through the left lateral mobile cartilage and secured to the inferior lateral laryngeal cartilage. Two other Prolene sutures were placed to anchor the mobile segment at anterior and right lateral position. The strap muscles were re-approximated with 4-0 Vicryl. The subplatismal layer was closed with 3-0 Vicryl. The skin was closed with 3-0 Prolene sutures.

No tracheobronchial, esophageal, or pharyngeal injuries were identified during laryngoscopy, bronchoscopy, and esophagoscopy. Timing of airway stabilization was around one hour from injuries. The time of definitive repair was around two hours from initial injury. The fracture of the left angle of mandible was repaired five days after initial injury. The patient received two doses of IV dexamethasone 10 mg every eight hours and IV antibiotics (clindamycin and ciprofloxacin) were used. Pulmonary contusions were managed conservatively.

Patient follow-up

**Figure 4:**
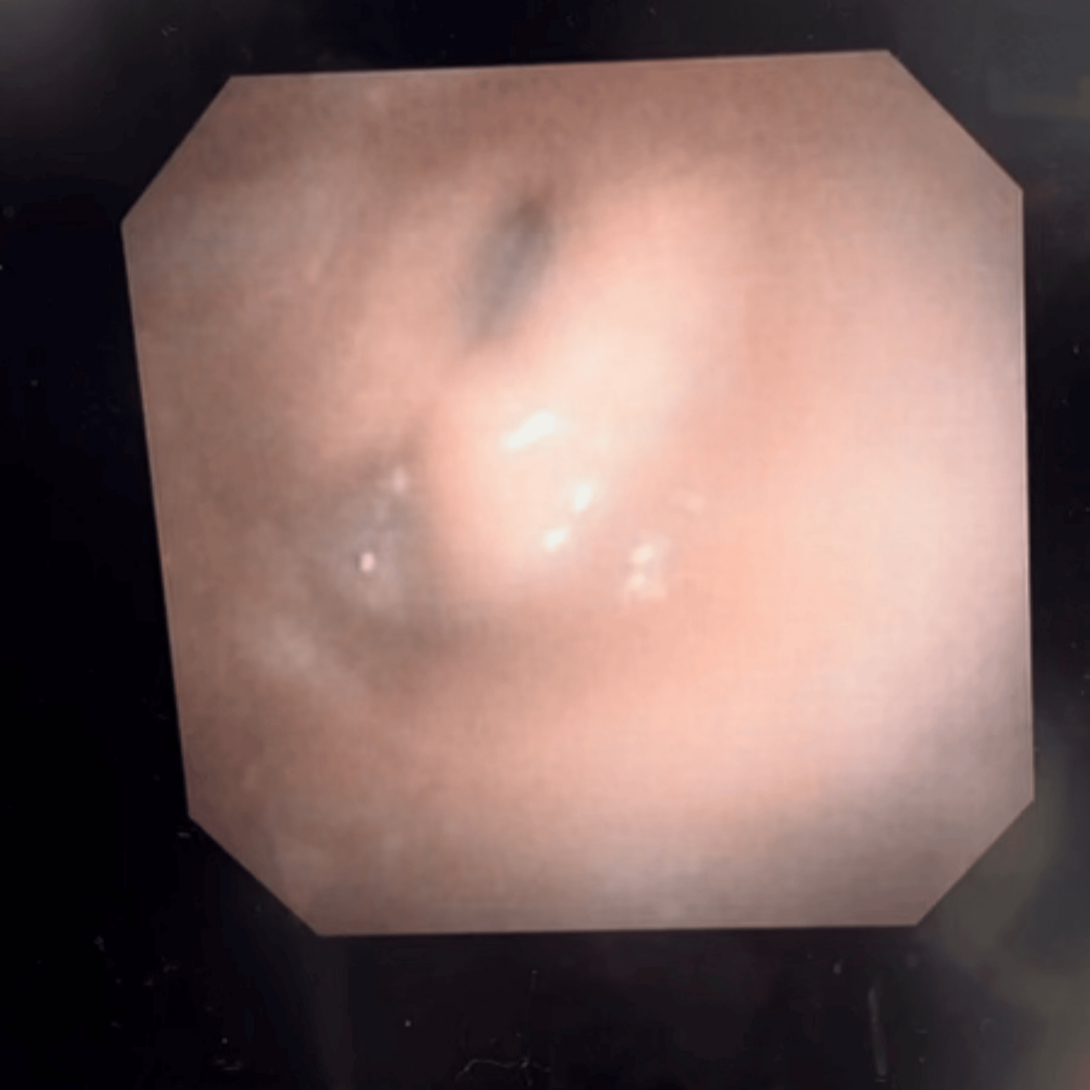
Laryngoscopy of the normal postoperative vocal cord adduction

**Figure 5:**
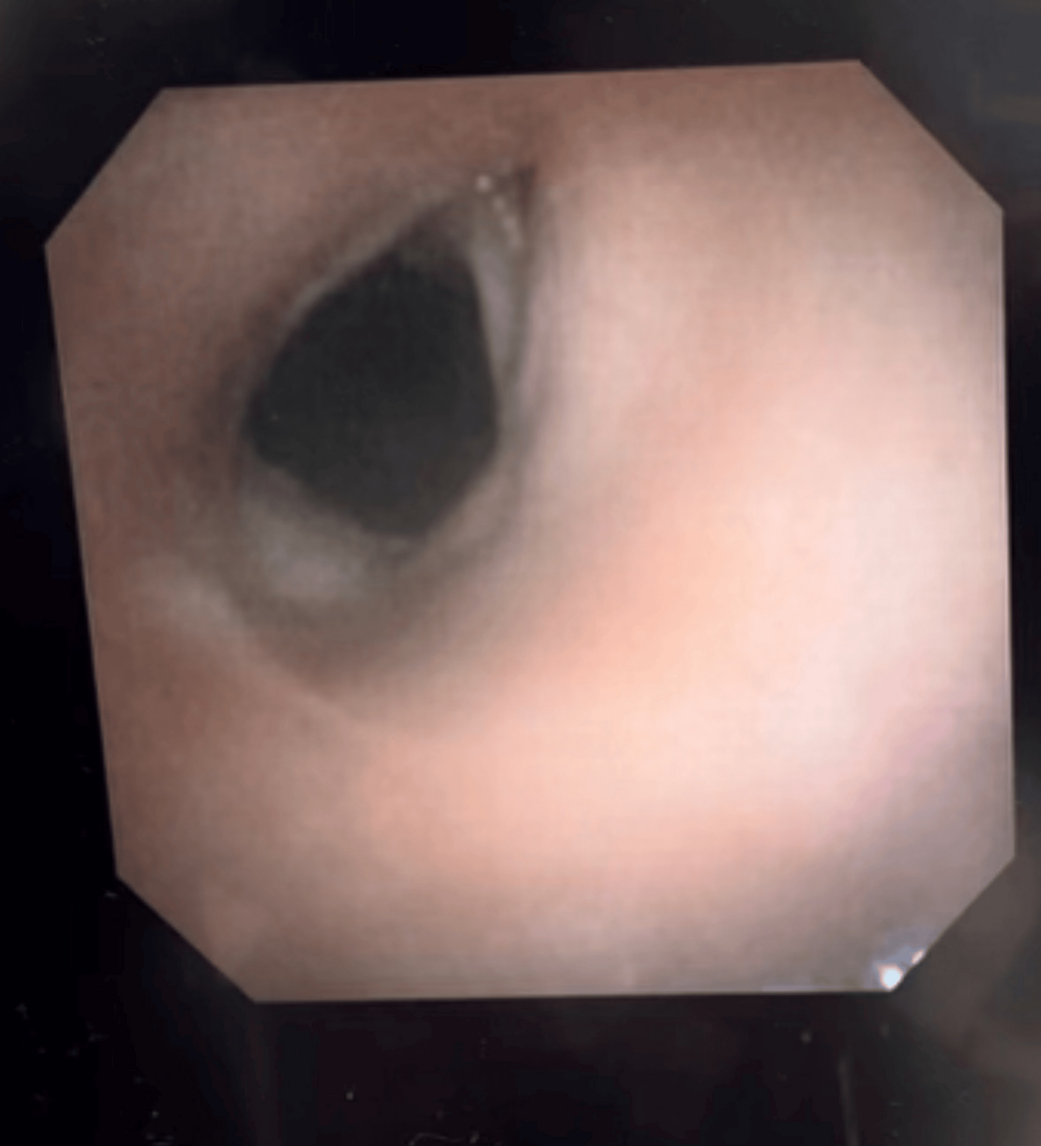
Laryngoscopy of the normal postoperative vocal cord abduction

At the one-month follow up, flexible endoscopy revealed normal bilateral movement and premorbid phonation (Figures 4, 5). 

**Figure 6:**
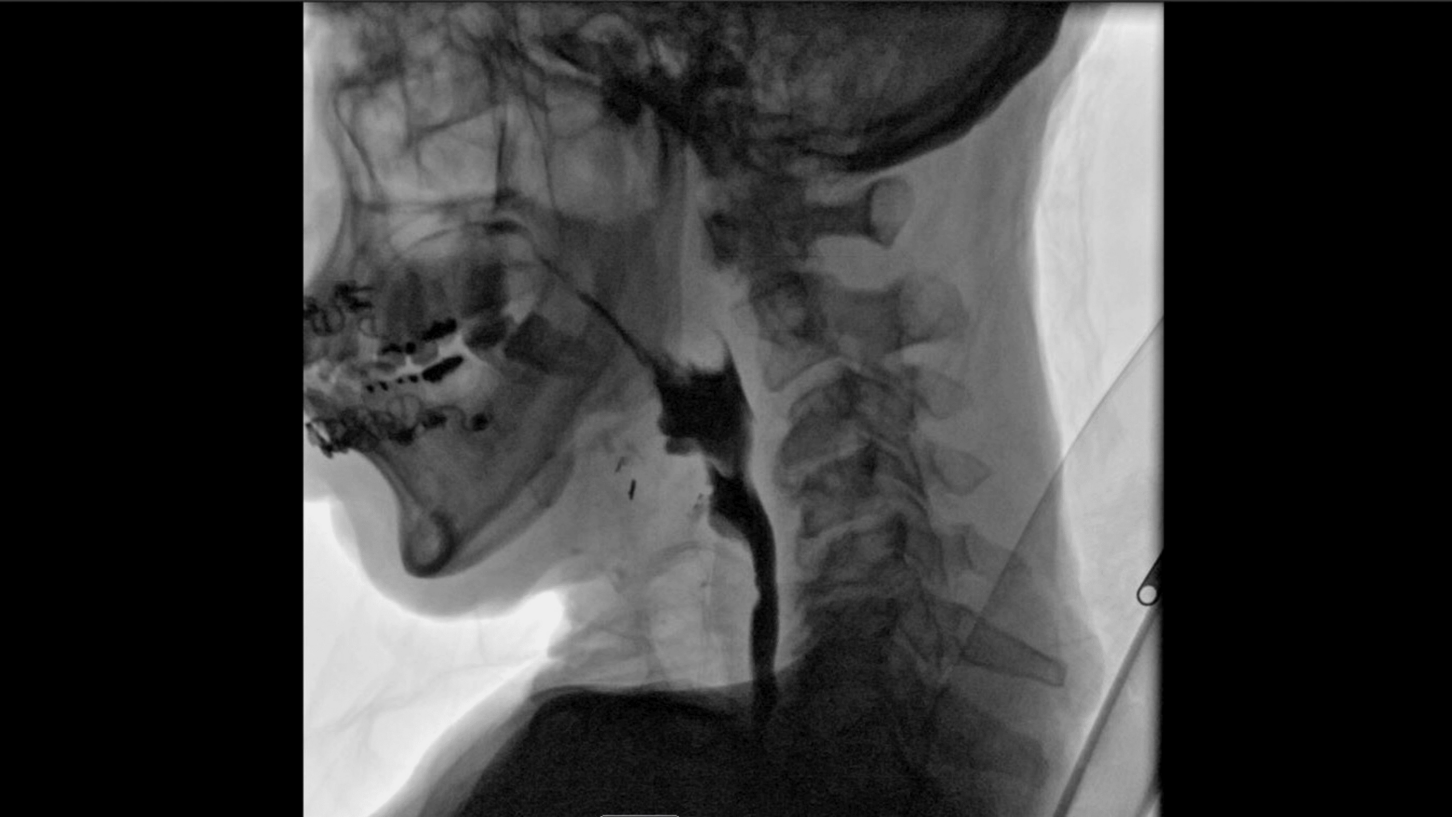
Modified barium swallow study

The swallow evaluation was normal (Figure 6) without any evidence of aspiration and the patient was decannulated without complication.

At the 12-month visit, the patient presented with no breathing or speech difficulties.

## Discussion

Laryngeal fracture is an uncommon but critical injury, most often associated with blunt trauma such as motor-vehicle collisions or direct blows [8]. Penetrating mechanisms, including gunshot wounds and stab wounds, can produce more severe and complex fractures due to direct tissue destruction. Laryngeal trauma with penetrative mechanisms has been associated with pre-hospital mortality rates between 20 to 40%, underscoring the vital importance of rapid airway assessment [9].

Our patient presented with classical features of significant laryngeal disruption: respiratory distress and diffuse subcutaneous emphysema. Although such findings raise suspicion, literature demonstrates that about 37% of patients have a delay in diagnosis, likely due to an absence of initial symptoms, lowering clinical concern [10]. This diagnostic challenge highlights the importance of maintaining a high index of suspicion in all penetrating neck injuries.

The primary objective in management is securing a safe, patent airway. While some authors advocate for careful endotracheal intubation under fiberoptic visualization, distorted anatomy - as in this case - often precludes safe intubation [11]. Surgical airways, including cricothyrotomy or tracheostomy, remain essential tools in such scenarios [2].

Once the airway is stabilized, attention first shifts to the acute care of penetrating neck, face, and upper torso injuries that may be life-threatening. Subsequently, and once stable, early surgical repair of displaced fractures has been shown to improve voice and swallowing outcomes [12]. Involvement of the anterior commissure is particularly significant because it is critical for vocal fold tension and glottic closure. Disruption may lead to long-term dysphonia if not meticulously repaired [13]. 

We believe that early anatomic repair and restoration of the specific structures contributed to a favorable outcome in our case. Some authors suggest placement of an endolaryngeal stent when significant mucosal injury is present or when anterior glottic stenosis is anticipated [14]. In this case, because the vocal fold mucosa was intact, we elected to proceed without a stent to minimize the risk of granulation and infection.

## Conclusions

Laryngeal fractures resulting from penetrating trauma necessitate immediate clinical recognition and decisive airway management to mitigate the risk of life-threatening respiratory compromise. The initial presentation can be deceptive, as occult cartilaginous damage may rapidly progress to complete obstruction. This case underscores that prioritizing a secure airway-often through early tracheostomy or expert intubation-is the critical first step in stabilization. Once the airway is protected, comprehensive diagnostic imaging and endoscopic evaluation are vital to delineate the extent of internal derangement before surgical intervention.

Optimizing long-term phonatory and respiratory outcomes depends heavily on early surgical repair and precise anatomic reconstruction. This is particularly crucial in injuries involving the anterior commissure, where meticulous realignment of the vocal folds is necessary to prevent synechiae formation and permanent dysphonia. As demonstrated by the patient’s excellent functional recovery, a multidisciplinary approach combining prompt intervention with rigid internal fixation ensures the restoration of the laryngeal framework. These findings reaffirm that timely anatomical reduction remains the gold standard for preserving the patient's quality of life in complex laryngeal trauma.
